# A scoping review of lesbian, gay, bisexual, transgender, queer, and intersex (LGBTQI+) people’s health in India

**DOI:** 10.1371/journal.pgph.0001362

**Published:** 2023-04-20

**Authors:** Venkatesan Chakrapani, Peter A. Newman, Murali Shunmugam, Shruta Rawat, Biji R. Mohan, Dicky Baruah, Suchon Tepjan

**Affiliations:** 1 Centre for Sexuality and Health Research and Policy (C-SHaRP), Chennai, India; 2 The Humsafar Trust, Mumbai, India; 3 Factor-Inwentash Faculty of Social Work, University of Toronto, Toronto, Ontario, Canada; 4 VOICES-Thailand Foundation, Chiang Mai, Thailand; Institute of Public Health Bengaluru, INDIA

## Abstract

Amid incremental progress in establishing an enabling legal and policy environment for lesbian, gay, bisexual, transgender and queer-identified people, and people with intersex variations (LGBTQI+) in India, evidence gaps on LGBTQI+ health are of increasing concern. To that end, we conducted a scoping review to map and synthesize the current evidence base, identify research gaps, and provide recommendations for future research. We conducted a scoping review using the Joanna Briggs Institute methodology. We systematically searched 14 databases to identify peer-reviewed journal articles published in English language between January 1, 2010 and November 20, 2021, that reported empirical qualitative, quantitative or mixed methods data on LGBTQI+ people’s health in India. Out of 3,003 results in total, we identified 177 eligible articles; 62% used quantitative, 31% qualitative, and 7% mixed methods. The majority (55%) focused on gay and other men who have sex with men (MSM), 16% transgender women, and 14% both of these populations; 4% focused on lesbian and bisexual women, and 2% on transmasculine people. Overall, studies reported high prevalence of HIV and sexually transmitted infections; multilevel risk factors for HIV; high levels of mental health burden linked to stigma, discrimination, and violence victimization; and non-availability of gender-affirmative medical care in government hospitals. Few longitudinal studies and intervention studies were identified. Findings suggest that LGBTQI+ health research in India needs to move beyond the predominant focus on HIV, and gay men/MSM and transgender women, to include mental health and non-communicable diseases, and individuals across the LGBTQI+ spectrum. Future research should build on largely descriptive studies to include explanatory and intervention studies, beyond urban to rural sites, and examine healthcare and service needs among LGBTQI+ people across the life course. Increased Indian government funding for LGBTQI+ health research, including dedicated support and training for early career researchers, is crucial to building a comprehensive and sustainable evidence base to inform targeted health policies and programs moving forward.

## Introduction

The right to the highest attainable standard of health is both universal and fundamental in international law [1]. This is enshrined in Article 12 [2] of the *Convention on Social*, *Economic*, *and Cultural Rights* and underlies United Nations Sustainable Development Goal 3 (SDG-3), which promises “Health for All” by 2030 and that “no one will be left behind.” This includes lesbian, gay, bisexual, transgender, queer identified, and people with intersex variations (LGBTQI+), who are entitled to the same standard of health as everyone else [3].

Despite the promise of the SDGs, evidence from across the globe suggests that LGBTQI+ health consistently lags behind that of the general public. Systematic and scoping reviews on health and healthcare access among LGBTQI+ people in high-income countries have shown that these populations continue to face disproportionate physical and mental health burdens in contrast to heterosexual populations [4–9]. For example, global reviews and large-scale studies have documented high levels of problematic alcohol use [10], sexualized drug use [11], mental health problems [4, 12], and high rates of HIV and other sexually transmitted infections (STIs) [13–15] among various LGBTQI+ subpopulations. Consistent with the minority stress model [16], many of these poor health outcomes are associated with societal stigma, discrimination, and violence, and systemic barriers in access to health services experienced by LGBTQI+ individuals [9, 17, 18].

Increasing recognition of health issues and disparities faced by LGBTQI+ people in the context of advances in LGBTQI+ rights movements globally have contributed to an evolving legal and policy environment that is becoming more supportive of LGBTQI+ rights, and more attuned to addressing LGBTQI+ health disparities and discrimination [19]. These advances in the recognition of LGBTQI+ rights have concomitantly contributed to increasing awareness of the need for research evidence to meaningfully implement this policy shift. Population-specific data are sorely needed to document gaps, disparities, and progress in LGBTQI+ health over time, as recognized by numerous bodies including the World Bank and UNDP; both have called for more attention and investment in research on LGBTQI+ health [20]. This trend is evident in India where the decriminalization of adult consensual same-sex relationships (2018) [21] and the enactment of the Transgender Persons Protection of Rights Act (2019) [22] have recently emerged in rapid succession. The latter act was designed, among other things, to support and promote the delivery of non-discriminatory and gender-affirmative health services to transgender people. Subsequently, India’s Ministry of Social Justice and Empowerment’s expert committee on issues related to transgender persons has called for research evidence to design interventions to improve the health of transgender people [23].

We are aware of no overview and thorough mapping of the evidence base on LGBTQI+ health in India. A few published reviews of LGBTQI+ health in India have focused on specific topics, such as HIV research among MSM or mental health issues among LGBTQI+ individuals [24–26]. To address the fragmented nature of current research knowledge, we conducted a scoping review to synthesize the evidence on LGBTQI+ health in India. The aim of this review was to characterize the breadth of published research on LGBTQI+ health in India and identify gaps in the evidence base, to provide recommendations for future research, and to synthesize existing evidence to inform health policies and interventions to advance LGBTQI+ health.

## Methods

We used the scoping review framework initially proposed by Arksey and O’Malley [27] and advanced by the Joanna Briggs Institute [28]. The key steps involved: (1) identifying the research questions; (2) identifying relevant studies; (3) study selection using a pre-defined set of inclusion and exclusion criteria; (4) charting the data; and (5) collating, summarizing and reporting the results.

### Research questions

The specific questions guiding this review were: (1) What are the peer-reviewed literature sources available on LGBTQI+ health in India?; (2) What health problems and conditions are reported among LGBTQI+ people?; and (3) What are the gaps in the available evidence on LGBTQI+ health in India? We conceptualized health problems and conditions broadly, including physical and mental health problems and conditions commonly addressed in the research with LGBTQI+ populations, such as HIV, depression, anxiety, and problematic alcohol use, as well as their social determinants, including stigma, discrimination, violence, and access to care.

### Identifying studies from academic databases

As the first comprehensive review of a broad range of health research among LGBTQI+ people across the vast geography and population of India, we limited our search to academic peer-reviewed journal articles. A literature search was conducted using the following academic databases: Medline, Education Resources Information Centre (ERIC), Applied Social Sciences Index and Abstracts (ASSIA), Public Affairs Information Service Index (PAIS Index), Bibliography of Asian Studies, EconLit, Education Source, Social Work Abstracts, Sociological Abstracts, PsychInfo, LGBTLife, Gender Studies, HeinOnline, ProQuest Thesis, Worldwide Political Science Abstracts, and Child and Adolescent Development. Search strings previously validated for LGBT+ populations [29] were used for identifying relevant articles. Search strings were customized to account for the unique syntax of each database surveyed (see S1 Appendix). We added relevant Indian LGBTQI+ terminology, including indigenous sexual role-based identity terms, such as kothi (feminine same-sex attracted males, primarily receptive sexual role), panthi (masculine and insertive role) and double-decker (both insertive and receptive role). We also searched for indigenous trans identities, such as hijras, thirunangai, jogappas, mangalmukhi, jogti hijras, and shivshaktis; however, as hijras was the only Indian language term used for trans identity in the article titles and abstracts, we used English language terms, such as trans men, trans women, trans person, and transgender. To delimit the results geographically, we added the term “India*” to all search strings. The searches from each database were documented, duplicates were eliminated, and citations were imported to Covidence (Veritas Health Innovation, Melbourne) for abstract and full-text screening.

### Study selection

Studies were selected according to pre-defined inclusion criteria. Studies must have been: 1) published between January 1, 2010 and November 20, 2021; conducted among LGBTQI+ people in India; 3) written in English; 4) peer reviewed; and 5) report primary data (qualitative, quantitative, or mixed methods). Two independent reviewers first screened the titles and abstracts for inclusion. In the case of discrepancies, a third reviewer was consulted to reach consensus. Full texts of potentially relevant articles were screened using a similar process. We selected the time frame to focus on recent articles relevant to current public health programs and policies in India, in order to identify extant research gaps and inform the future research agenda. Additionally, the third phase of India’s National AIDS Control Programme (NACP-III), launched in late 2009, explicitly addressed targeted HIV interventions for men who have sex with men and transgender women, which brought national attention to the health issues of sexual and gender minority populations.

### Charting, collating and summarizing the results

The following data were extracted for analysis: year of publication, study location, sample size, study population, objectives, design, methodology (qualitative, quantitative or mixed methods) and key findings. We summarized the results using frequencies, and thematic analysis and synthesis [28]. Studies were grouped by key themes that emerged from the synthesis: prevalence of HIV and STIs, and risk factors; stigma, discrimination and violence, and health impact; access to health services; interventions to improve health outcomes among LGBTQI+ populations; new HIV prevention technologies and their acceptability; and under-represented LGBTQI+ populations.

## Results

### Study selection

**Fig 1:**
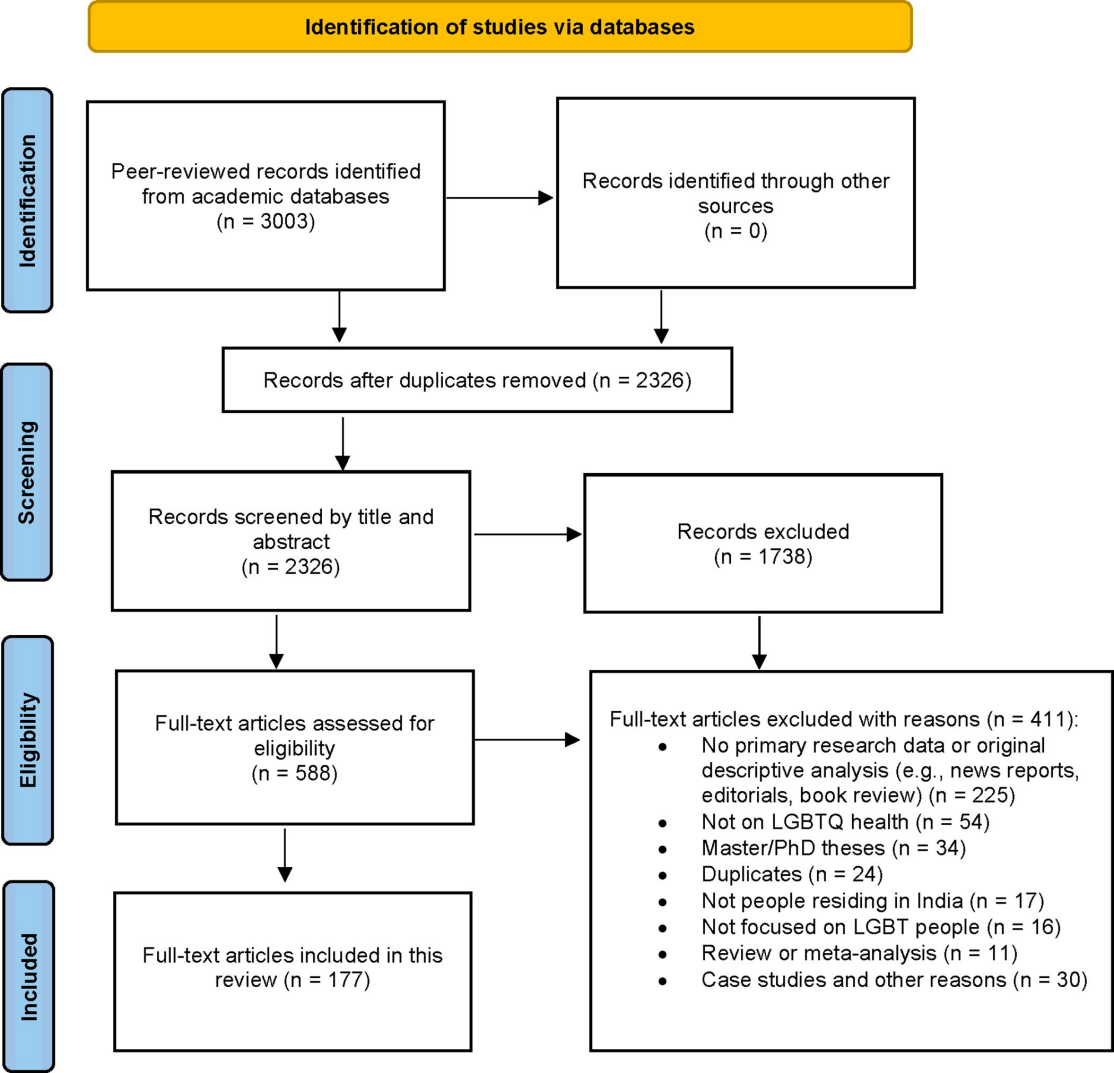
PRISMA flowchart of study selection.

The search strategy yielded 2,326 sources after removing duplicates. Screening of the titles and abstracts yielded 588 articles included in full-text review. Of these, 177 peer-reviewed articles met the *a priori* eligibility criteria and were included in the scoping review (Fig 1). We extracted study characteristics and key findings for the included articles (Table 1).

**Table 1:** Study characteristics and themes of inclusion (n = 177).

Author(s)	Year	N	Focal Population(s)						Methods			Themes					Main Funding Source
									Quantitative	Qualitative	Mixed	HIV/STI	Stigma/Discrimination	Access to Services	Interventions^a^	New Prevention Tech	
			GBMSM	TGW	LBWSW	TGM	Ppl with intersex var.	Other									
Solomon et al. [30]	2010	721	X						X			X					Fogarty International Center, United States; NIH^e^, United States
Ghosh et al. [31]	2011	32	X						X			X					No funding information provided
Sahastrabuddhe et al. [32]	2012	84		X					X			X					ICMR^f^, India; NIAID^g^ United States; NIH
Ghosh et al. [33]	2012	26	X						X			X					NACO^h^, India
Solomon et al. [34]	2015	12,022	X						X			X					NIH; John Hopkins Centre for AIDS Research, United States
Mayer et al. [35]	2015	307	X						X			X					Indo-US grant
Hernandez et al. [36]	2016	300	X						X			X					Indo-US grant
Aggarwal et al. [37]	2016	52	X						X			X					No funding
Solomon et al. [38]	2016	12,022	X						X			X					NIH; Elton John AIDS Foundation
Raghavendran et al. [39]	2017	300	X						X			X					Indo-US grant
Hussain et al. [40]	2018	277	X						X			X					No funding information provided
Gupte et al. [41]	2011	2,633	X	X					X			X					Bill & Melinda Gates Foundation
Clipman et al. [42]	2020	4,994	X						X			X					NIH; John Hopkins Centre for AIDS Research, United States
Haldar et al. [43]	2020	2,584	X						X			X					No funding
Hernandez et al. [44]	2021	302	X						X			X					Indo-US grant
Palakkal et al. [45]	2020	560	X						X			X					No funding
Prabhu et al. [46]	2021	1,639	X						X			X					NIH; John Hopkins Centre for AIDS Research, United States; Elton John AIDS Foundation
Patel et al. [47]	2021	20,002	X						X			X					NIH; Elton John AIDS Foundation
Kumta et al. [48]	2010	831	X						X			X					Mumbai District AIDS Control Society, India
Phillips et al. [49]	2010	357	X						X			X					Bill & Melinda Gates Foundation
Solomon et al. [50]	2010	781	X								X	X					Indo-US grant
Setia et al. [51]	2010	511	X						X			X					CIHR^i^, Canada
Gutierrez et al. [52]	2010	4,321	X						X			X					Bill & Melinda Gates Foundation
Lorway et al. [53]	2010	120	X						X			X					CIHR
Mimiaga et al. [54]	2011	210	X						X			X					NIH; NIAID
Hendriksen et al. [55]	2011	5	X							X		X					NIAID
Hemmige et al. [56]	2011	676	X						X			X					American Foundation for AIDS Research; Centers for Disease Control and Prevention (CDC) Global AIDS Program (GAP), United States
Lorway et al. [57]	2011	--	X							X		X					CIHR
Thomas et al. [58]	2012	210	X						X			X					NIH; NIAID
Tomori et al. [59]	2018	47	X							X		X					NIH; John Hopkins Centre for AIDS Research, United States
Wilkerson et al. [60]	2018	433	X						X			X					Indo-US grant
Srivastava et al. [61]	2019	10	X							X		X					Asian Network to Address Masculinities; The University Grants Commission, India
Mimiaga et al. [62]	2013	150	X						X			X					Fenway Institute, United States; NIH
Saggurti et al. [63]	2013	2,399	X	X					X			X					Bill & Melinda Gates Foundation
Chakrapani et al. [64]	2013	88	X							X		X					Department for International Development, United Kingdom
Ramanathan et al. [65]	2013	1618	X						X			X					Bill & Melinda Gates Foundation
Narayanan et al. [66]	2013	483	X						X			X					Bill & Melinda Gates Foundation
Kumar et al. [67]	2014	3,229	X						X			X					Bill & Melinda Gates Foundation
Yadav et al. [68]	2014	3,880	X						X			X					Bill & Melinda Gates Foundation
Ramesh et al. [69]	2014	1,608	X						X			X					Bill & Melinda Gates Foundation
Mitchell et al. [70]	2014	595	X						X			X					Wellcome Trust; Bill & Melinda Gates Foundation
Closson et al. [71]	2014	32	X							X		X					Indo-US grant
Ramanathan et al. [72]	2014	1,305	X	X					X			X					Bill & Melinda Gates Foundation
Godbole et al. [73]	2014	4,682	X						X			X					Department of AIDS Control, Ministry of Health and Family Welfare, Government of India
Mitchell et al. [74]	2014	320	X						X			X					Wellcome Trust; Bill & Melinda Gates Foundation
Saha et al. [75]	2015	227	X						X			X					NACO
Saha et al. [76]	2015	243	X						X			X					NACO
Ramakrishnan et al. [77]	2015	3,833	X						X			X					Bill & Melinda Gates Foundation
Mahapatra et al. [78]	2015	1,237	X						X			X					NACO
Shaw et al. [79]	2016	456	X	X					X			X					Bill & Melinda Gates Foundation
Tomori et al. [80]	2016	12,151	X								X	X					NIH; Johns Hopkins Center for AIDS Research, United States
Sinha et al. [81]	2017	90		X					X			X					No funding
Banik et al. [82]	2019	72	X							X		X					Indiana University, Bloomington, United States; National Institute on Drug Abuse, United States
Deshpande et al. [83]	2015	689	X						X			X					Bill & Melinda Gates Foundation
Chakrapani et al. [84]	2015	151	X	X							X	X					Department for International Development, United Kingdom
Dodge et al. [85]	2016	72	X								X	X					Indiana University, Bloomington, United States; NIH
Willie et al. [86]	2017	299		X					X			X					ICMR
Ferguson et al. [87]	2016	30	X	X						X		X					Yale University, United States
Banik et al. [88]	2014	36	X							X		X					NIH; Cleveland State University, United States
Wilkerson et al. [89]	2019	449	X	X					X			X					Indo-US grant
Bhambhani et al. [90]	2021	4,321	X						X			X					NIH
Sudharshan et al. [91]	2020	33	X						X			X					No funding
Safren et al. [92]	2021	608	X						X			X					NIH
Kumar et al. [93]	2020	23,081	X						X			X					NACO
Rajan et al. [94]	2020	3,325		X					X			X					No funding
Sivasubramanian et al. [95]	2011	150	X						X				X				Fenway Health, United States
Logie et al. [96]	2012	200	X						X				X				CIHR; SSHRC^j^; CIDA^k^
Shaw et al. [97]	2012	543	X	X					X				X				Bill & Melinda Gates Foundation
Tomori et al. [98]	2018	484	X							X			X				NIMH^l^; Johns Hopkins Center for AIDS Research, United States
Tomori et al. [99]	2018	11,771	X						X				X				NIH; Johns Hopkins Center for AIDS Research, United States
Thaker et al. [100]	2018	225	X	X					X				X				National University, Singapore
Chakrapani et al. [101]	2019	300		X					X				X				ICMR
Thompson et al. [102]	2013	39	X							X			X				CIHR
Elouard et al. [103]	2013	11	X							X			X				No funding information provided
Maroky et al. [104]	2015	51	X						X				X				No funding
Mimiaga et al. [105]	2015	55	X							X			X				Indo-US grant
Tomori et al. [106]	2016	12,355	X								X		X				NIH; Johns Hopkins Center for AIDS Research, United States
Tomori et al. [107]	2016	363	X							X			X				NIH; Johns Hopkins Center for AIDS Research, United States
Chakrapani et al. [108]	2017	600	X	X					X				X				ICMR
Ganju et al. [109]	2016	68		X						X			X				Bill & Melinda Gates Foundation
Chakrapani et al. [110]	2017	600	X	X					X				X				ICMR
Kalra et al. [111]	2013	50		X					X				X				No funding information provided
Lorway et al. [112]	2013	70	X							X			X				Bill & Melinda Gates Foundation
Manian [113]	2014	10	X	X						X			X				No funding
Chakrapani et al. [114]	2017	300		X					X				X				ICMR
Chakrapani et al. [115]	2018	40	X							X			X				Wellcome Trust / DBT India Alliance Senior Fellowship
Rao et al. [116]	2018	227						X^b^	X				X				Psi Chi, the international honor society in psychology, United States
Pandya [117]	2010	250	X							X			X				No funding information provided
Dutta et al. [118]	2019	41		X						X			X				CIHR; SSHRC
Bowling et al. [119]	2019	58	X	X	X					X			X				Bill & Melinda Gates Foundation
Rao et al. [120]	2020	170						X^b^	X				X				Psi Chi, the international honor society in psychology, United States
Li et al. [121]	2017	487	X	X							X		X				Indo-US grant
Chavada et al. [122]	2021	100		X					X				X				No funding
Bhattacharya et al. [123]	2020	98	X	X					X				X				NIH; Department of Geography, University of Connecticut, United States
Dhabhar et al. [124]	2020	112	X		X				X				X				No funding
Banerjee et al. [125]	2020	10		X						X			X				No funding information provided
Azhar et al. [126]	2021	16						X^c^			X		X				Minority Fellowship Program; University of Chicago Center for the Study of Gender and Sexuality, United States
Srivastava et al. [127]	2021	20		X						X			X				No funding information provided
Prabhu et al. [128]	2020	1,454	X						X				X				NIH; Elton John AIDS Foundation
Arvind et al. [129]	2021	20		X							X		X				No funding information provided
Thirunavukkarasu et al. [130]	2021	235	X						X				X				No funding
Sharma et al. [131]	2020	296	X	X	X						X		X				Department of Science and Technology, India; Indian Institute of Technology, Gandhinagar
Dhaor [132]	2021	21	X	X							X		X				No funding
Safren et al. [133]	2021	608	X						X				X				National Institute of Mental Health, United States
Pufahl et al. [134]	2021	184						X^c^	X				X				US Consulate, Mumbai, India
Majumder et al. [135]	2020	37		X					X				X				No funding
Joshi et al. [136]	2021	33		X					X				X				World Pranic Healing Foundation, India
Jesus et al. [137]	2020	23		X							X		X				Sanford School of Public Policy; Duke Global Health Institute, Duke University, United States
Sharma et al. [138]	2020	207	X								X		X				No funding
Sartaj et al. [139]	2021	50		X					X				X				No funding
Srivastava et al. [140]	2021	3,548	X	X					X				X				No funding
Jethwani et al. [141]	2014	124	X						X				X				No funding
Singh et al. [142]	2018	15	X						X				X				No funding information provided
Mogasale et al. [143]	2010	--	X	X					X					X			Bill & Melinda Gates Foundation
Chakrapani et al. [144]	2011	38	X	X						X				X			The International Treatment Preparedness Coalition (ITPC); European Union (EU)/The Humanist Institute for Development Cooperation (HIVOS)
Gurung et al. [145]	2011	8,9621	X	X					X					X			Bill & Melinda Gates Foundation
Woodford et al. [146]	2012	132	X						X					X			Canada Research Chairs Program; SSHRC
Beattie et al. [147]	2012	90	X	X						X				X			Bill & Melinda Gates Foundation
Pina et al. [148]	2018	300	X	X					X					X			The Global Health Scholarship from the Rosenbluth Fund, Einstein’s Global Health Center; NIH
Patel et al. [149]	2018	4,179	X						X					X			NIH
Samuel et al. [150]	2018	212		X					X					X			No funding information provided
Ramesh et al. [151]	2015	3,229	X						X					X			Bill & Melinda Gates Foundation
Mehta et al. [152]	2015	1,146	X						X					X			NIH; Johns Hopkins University Center for AIDS Research, United States
McFall et al. [153]	2016	503	X						X					X			NIH; Johns Hopkins University Center for AIDS Research, United States
Singh et al. [154]	2014	94		X						X				X			Global Fund to Fight AIDS, Tuberculosis and Malaria
Woodford et al. [155]	2016	47	X	X						X				X			CIHR; Canada Research Chairs Program
Acharya et al. [156]	2021	18	X	X					X					X			No funding
Pandya et al. [157]	2021	12		X						X				X			No funding information provided
Pollard et al. [158]	2021	28	X	X						X				X			U.S. President’s Emergency Plan for AIDS Relief (PEPFAR); USAID^m^
Achuthan et al. [159]	2021	51						X^d^		X				X			Ford Foundation
Kulkarni et al. [160]	2021	6		X						X				X			No funding
Kurian et al. [161]	2021	40		X						X				X			No funding
Ghosh et al. [162]	2020	22		X						X				X			NACO
Tom et al. [163]	2021	22		X						X				X			No funding information provided
Ranade et al [164]	2013	25						X^d^		X				X			No funding information provided
Snyder et al. [165]	2012	298	X						X						X		American Foundation for AIDS Research (amFAR); NIH
Thomas et al. [166]	2012	55	X							X					X		Indo-US grant
Safren et al. [167]	2014	96	X						X						X		Indo-US grant
Shaikh et al. [168]	2016	268		X					X						X		Global Fund to Fight AIDS, Tuberculosis, and Malaria
Solomon et al. [169]	2016	10,000	X						X						X		NIH; Elton John AIDS Foundation
Thomas et al. [170]	2017	75	X							Xc					X		Indo-US grant
Mimiaga et al. [171]	2017	100	X						X						X		Indo-US grant
Roy et al. [172]	2015	16	X							X					X		No funding information provided
Chakrapani et al. [173]	2020	119	X						X						X		ICMR
Chakrapani et al. [174]	2020	459	X						X						X		Postgraduate Institute of Medical Education and Research (PGIMER), India
Patel et al. [175]	2020	244	X						X						X		NIH
Eisingerich et al. [176]	2012	128	X						X							X	Bill & Melinda Gates foundation
Uthappa et al. [177]	2018	400	X	X					X							X	ICHHA foundation, India
Chakrapani et al. [178]	2012	82	X							X						X	CIHR; SSHRC; Canada Research Chairs program
Chakrapani et al. [179]	2013	82	X							X						X	CIHR; Canada Research Chairs program; Canada Foundation for Innovation
Newman et al. [180]	2014	400	X						X							X	CIHR; Canada Research Chairs program; Canada Foundation for Innovation
McClarty et al. [181]	2015	379						X^d^	X							X	CIHR; Canadian HIV Vaccine Initiative
Chakrapani et al. [182]	2015	71	X							X						X	CIHR; Canada Research Chairs Program
Ramanaik et al. [183]	2015	50						X^d^		X						X	The International Infectious Disease and Global Health Training Program, University of Manitoba, Canada; CIHR
Mitchell et al. [184]	2016	--	X						X							X	Bill & Melinda Gates Foundation; NIH
Chakrapani et al. [185]	2017	71	X							X						X	CIHR
Schneider et al. [186]	2012	387	X						X							X	American Foundation for AIDS Research; NIH
Chakrapani et al. [187]	2020	44		X						X						X	CIHR; Canada Research Chairs Program
Chakrapani et al. [188]	2021	197	X						X							X	CIHR; Canada Foundation for Innovation
Belludi et al. [189]	2021	8,621	X						X							X	National Institute on Drug Abuse, United States; NIMH
Chakrapani et al. [190]	2021	197	X						X							X	CIHR
Rao et al. [191]	2020	56	X	X						X						X	World Health Organization
Kazemian et al. [192]	2020	--	X						X							X	NIH; Harvard University Center for AIDS Research, United States
Chakrapani et al. [193]	2021	360		X					X							X	ViiV Health Care
Bowling et al. [194]	2018	67			X						X						Indiana University’s School of Public Health, Bloomington, United States
Apoorva et al. [195]	2016	8			X					X							No funding information provided
Chithrangathan [196]	2018	1			X					X							No funding information provided
Banerjea [197]	2015	8			X					X							No funding information provided
Bowling et al. [198]	2016	20			X					X							Indiana University’s School of Public Health, Bloomington, United States
Bowling et al. [199]	2018	18			X					X							Indiana University’s School of Public Health, Bloomington, United States
Srivastava [200]	2020	25			X					X							Indian University Grant Commission Doctoral Fellowship
Bowling et al. [201]	2019	58	X	X	X					X							Bill & Melinda Gates Foundation
Bowling et al. [202]	2019	33	X	X	X					X							Bill & Melinda Gates Foundation
Chakrapani et al. [203]	2021	27				X				X							DBT/Wellcome Trust India Alliance Senior Fellowship
Majumder et al. [204]	2021	120		X		X			X								No funding
Das [205]	2020	29					X			X							No funding information provided
Joseph et al. [206]	2017	205					X		X								No funding information provided

Note: Terminologies for focal populations are derived from original sources, with indigenous sexual and gender identities in italics. GBMSM = Gay, bisexual and other men who have sex with men; TGW = Transgender women; LBWSW = Lesbian, bisexual, and other women who have sex with women; TGM = Transgender men; Ppl with intersex var. = People with intersex variations.

^a^Interventions to improve health outcomes

^b^Sexual gender minority

^c^Gender non-confirming

^d^Health service providers

^e^National Institute of Health (NIH), United States

^f^Indian Council of Medical Research (ICMR), India

^g^National Institute of Allergy and Infectious Diseases (NIAID), United States

^h^National AIDS Control Organization (NACO), India

^i^Canadian Institutes of Health Research (CIHR), Canada

^j^Social Sciences and Humanities Research Council (SSHRC), Canada

^k^Canadian International Development Agency (CIDA), Canada

^l^National Institute of Mental Health (NIMH), United States

^m^United States Agency for International Development, United States

### Study characteristics

**Fig 2:**
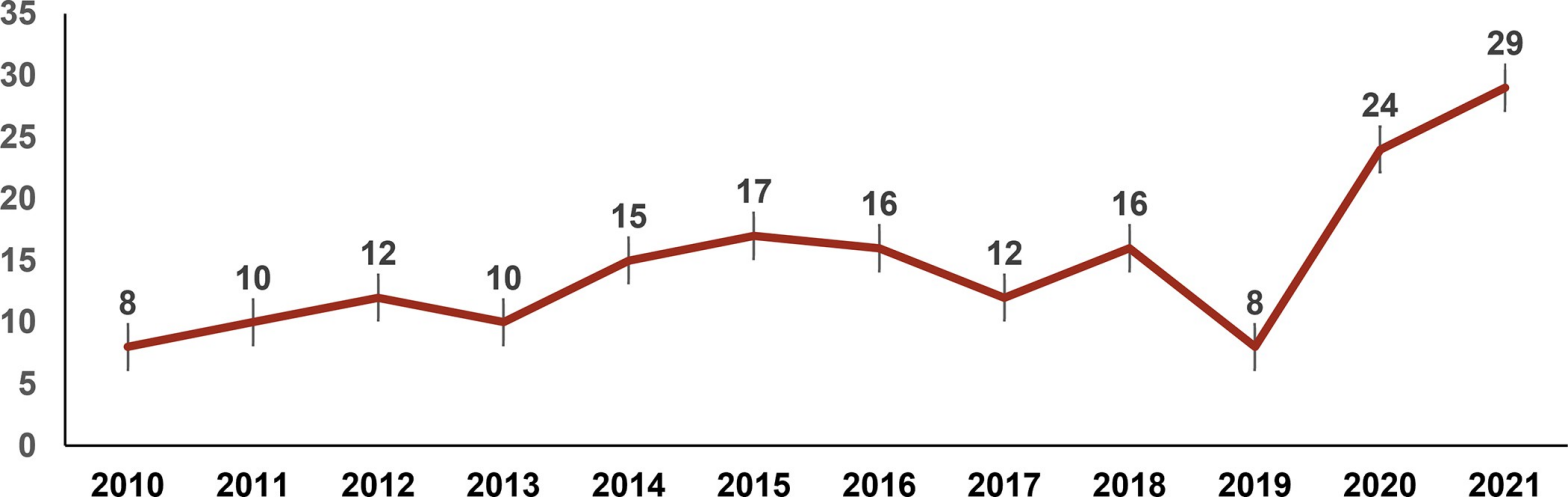
Distribution of peer-reviewed articles by year of publication (N = 177).

**Fig 3:**
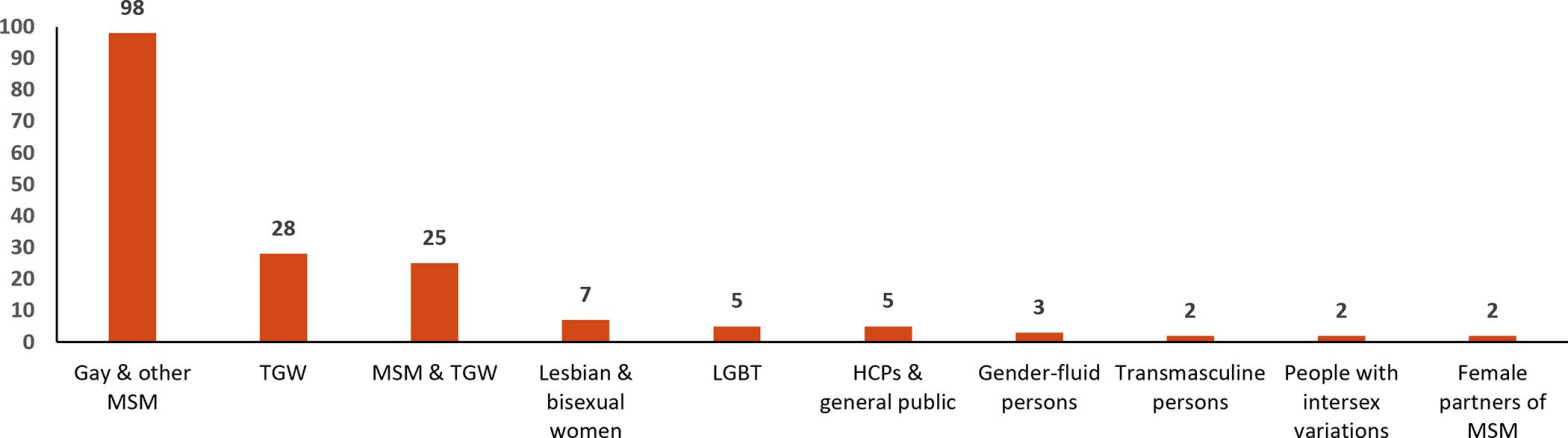
Distribution of focal populations in the peer-reviewed articles (N = 177). HCP, healthcare professional; LGBT, lesbian, gay, bisexual and transgender; MSM, men who have sex with men; TGW, transgender women.

Of the 177 articles, the majority (59%; n = 105) were published from 2016 onward (Fig 2). In terms of methodology, 62% were quantitative, 31% qualitative, and 7% mixed methods studies. A majority (55%; n = 98) of studies were conducted among MSM, 16% (n = 28) among TGW, and 14% (n = 25) among both MSM and TGW (Fig 3). Seven studies (4%) were conducted among lesbian or bisexual women, five (3%) among LGBTQI+ people as a whole, and two each among transmasculine people, and people with intersex variations.

Nearly half (47%; n = 84) of the studies were conducted in four (of 28) Indian states—Maharashtra (n = 30), Tamil Nadu (n = 23), Karnataka (n = 19) or Andhra Pradesh (n = 12), with the majority of these in state capitals—Mumbai, Chennai, Bangalore, or Hyderabad. Over a third (36%; n = 65) of the studies were conducted in multiple Indian states.

Overall, 77% of studies (n = 137/177) reported sources of funding support, and 12% (n = 21) reported not receiving any specific funding; 11% (n = 19) did not report sources of funding. Of those studies that reported a funding source, the majority (72%; n = 99/137) were foreign sources (largely from the U.S. National Institutes of Health [NIH] and the Bill and Melinda Gates Foundation); 12% (n = 17) were Indo-U.S. collaborative research projects funded jointly by the Indian Council of Medical Research (ICMR) and NIH. Twenty studies (15%) received primary funding from the government of India (Indian Council of Medical Research [ICMR] and the National AIDS Control Organization [NACO]) and other Indian institutions.

### HIV/STI prevalence and risk factors

**Fig 4:**
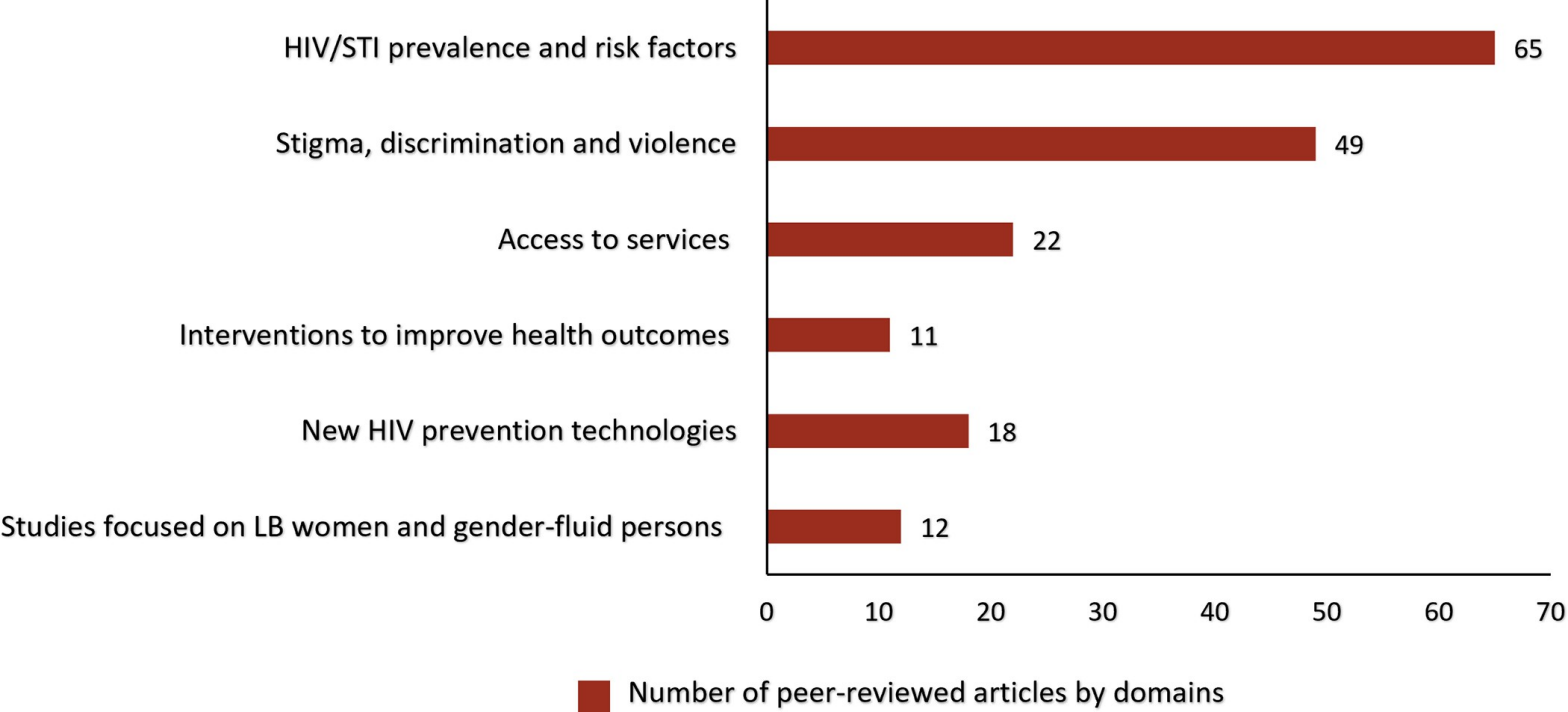
Distribution of peer-reviewed articles by domains (N = 177). LB, lesbian and bisexual.

Thirty-seven percent (n = 65) of the articles focused on reporting STI/HIV prevalence estimates [30–47] and correlates of HIV-related risk behaviors [48–94] among MSM and TGW (Fig 4). In the 18 studies [30–47] that reported HIV and STI prevalence estimates among MSM and TGW, nine [31–33, 37, 39–42, 45] were conducted in clinical settings, six [30, 34, 35, 38, 43, 46] in community settings, and three [36, 44, 47] in both clinical and community settings. Of these 18 studies, eight [30, 34, 35, 37, 40, 43, 45, 46] reported HIV/STI prevalence and risk factors among MSM, three [36, 39, 44] human papillomavirus (HPV) prevalence among MSM living with HIV, and three [31, 33, 45] reported prevalence of perianal dermatoses, HPV and other STIs (such as syphilis, chlamydia and gonorrhea) among MSM. Two studies [38, 47] reported correlates of HIV incidence among MSM, with one study each reporting Hepatitis C prevalence among MSM living with HIV [42], and one study the prevalence of herpes [45].

Overall, HIV prevalence among MSM ranged from 3.8% to 23.0% across different study sites. Among MSM, HPV/genital warts (23.0% to 95.0%), syphilis (0.8% to 11.9%), HSV/genital herpes (7.1 to 32.0%), and genital molluscum contagium (9.6%) were the most commonly reported STIs [30, 31, 33, 34, 36, 37, 39, 40, 42, 45]. One study [42] reported Hepatitis-C prevalence among MSM as 1.3%. Syphilis rates tended to be higher among single MSM (8.3%) than married MSM (1.0%) [35]. In a study [32] conducted among 84 TGW who attended STI clinics in Pune, HIV prevalence was 45.2%.

Forty-seven articles [48–94] reported correlates of HIV-related risk among MSM and TGW. Among MSM, significant correlates of HIV risk behaviors/indicators such as condomless sex [48–50, 58, 64, 77, 78, 92, 93], infrequent HIV testing [60, 65, 72, 74], and HIV/STI positivity [48, 51, 55, 79] were low literacy and unemployment [48, 76, 77], alcohol and/or drug use [54, 60, 64, 65, 79, 90, 93], engagement in sex work [49, 60, 61, 65, 67, 68, 75, 76, 78], higher number of male sexual partners [48–50, 53, 56, 72, 74], early age of sexual debut [93], and low HIV risk perception [60, 65, 72, 74]. Six of the 47 articles included data on TGW; five of these [79, 81, 84, 87, 89] did not provide details on correlates of HIV risk behaviors, with one study [94] reporting that having a male regular partner was associated with HIV seropositivity.

### Stigma, discrimination, and violence, and health impacts

Over one-fourth of the articles (27%; n = 48) [95–142] reported on stigma, discrimination, violence, and their associations with physical and mental health. Among these, 16 articles focused on stigma-related aspects of LGBTQI+ health [96, 100–102, 109, 110, 112, 114, 119, 124–126, 129, 132, 137, 140], 3 on violence [97, 103, 118], 17 on mental health and its correlates, such as quality of life [95, 99, 105, 106, 108, 111, 115, 123, 127, 128, 130, 131, 135, 136, 138, 139, 142], two on resilience [122, 133] and one article each on coping skills [141] and promoting LGBTQI+ acceptance [134]. Three articles reported on stress [116], perceived psychological impact [120] and violence [121] associated with Section-377 of the Indian Penal Code, which until September 2018 criminalised adult consensual same-sex relationships.

Several studies highlighted various types of stigma and discrimination experienced by MSM and TGW, which include perceived stigma, felt normative stigma, HIV-related stigma, family-enacted stigma, gender non-conformity stigma, and internalized stigma [96, 100, 101, 109, 124–126, 129, 132, 138], gender discrimination, workplace discrimination [137, 139] and polyvictimization [140]. Perpetrators of discrimination and violence against MSM and TGW, including those living with HIV, included peers, sexual partners, family members, healthcare providers, and police [98, 102, 103, 109, 112, 118, 119, 129, 130, 137, 139]. Fear of discrimination and suboptimal care [112] or refusal of care [109] prevented some persons from disclosing their sexual or gender identity to healthcare providers.

Fifteen studies [98, 99, 107–109, 112, 115, 125, 127–130, 137, 139, 140] indicated that stigma and discrimination contribute to depression and other negative mental health outcomes, such as suicidal ideation or attempts, among sexual and gender minorities. Two studies documented a high prevalence of mental health issues among MSM: depression (29% to 45%), anxiety (24% to 40%), suicidal ideation (45% to 53%), suicide attempts (23%), substance abuse (28%) including alcohol dependence (15% to 22%) [95, 130]. Similarly, among TGW, high levels of depression (43%), problematic alcohol use (37%) [108], anxiety (39%), depression (21%), suicide risk (75.8%) [136] and violence (52%) [139] were reported. Three studies with MSM [99, 108, 109, 115] and one with MSM and TGW [108] reported psychosocial syndemics, that is, co-occurring psychosocial conditions such as problematic alcohol use and internalized homonegativity, and their synergistic impact on HIV risk. The COVID-19 pandemic was also addressed as exacerbating psychological distress among LGBTQI+ people [125, 131].

Several studies addressed resilience, coping, and social support. A few studies documented various types of social support and other resilience resources available to MSM and TGW [107, 109, 117], with one study reporting moderate or high levels of resilience among 72% of TGW [122]. In terms of coping with adversity, MSM and TGW reported supportive roles of peers, NGOs [109], family, friends and partners [107], and gharanas (‘clans’ or houses of hijra-identified trans people) [127]. Some MSM and TGW reported strategies to prevent violence, discrimination and psychological distress, which included bribing police, running away from unsafe places and persons, and negotiating condom use during forced sex encounters [109], hiding sexual identities [103], denial [123], and behavioral disengagement [141]. One study documented positive coping strategies among older transgender people, such as spirituality, hope, and acceptance of gender dissonance [125]. In a few studies, social support and resilient coping strategies were identified as predictors of HIV risk [108] or mediators and moderators of the effects of discrimination on HIV risk or depression [110]. A resilience-based psychosocial intervention that integrated counselling was found to be effective in reducing HIV risk among MSM, with self-esteem and depressive symptoms mediating this effect [133]. A community-based theatre intervention was identified as effective in improving positive attitudes and knowledge, and promoting acceptance and solidarity towards LGBTQI+ communities among young adult heterosexual audiences [134].

### Access to services: HIV/STIs and gender-affirmative procedures

In total, 22 studies [143–164]—10 quantitative [143, 145, 146, 148–153, 156] and 12 qualitative [144, 147, 154, 155, 157–164]—investigated access to HIV/STI services, gender transition services, and other clinical services. Four of these studies focused on HIV testing [145, 148, 150, 154] and four [144, 148, 156, 162] on antiretroviral treatment (ART) access and uptake among MSM and TGW living with HIV. Two studies [152, 153] addressed the HIV care continuum and linkages to care, three [147, 157, 158] challenges in accessing HIV testing, treatment and care services among MSM and TGW. Five studies focused on access to healthcare and support services for TGW: access to gender transition services [154], barriers to dental [150] and eye care [160], gender-affirmative technologies [159], and welfare schemes for TGW [161].

In relation to HIV testing among MSM, quantitative studies [146, 149, 151] reported that a majority of those recruited through community-based organizations (CBOs) or public sex environments were tested for HIV (61% to 86%) [146, 151], in contrast to MSM recruited through online social networking sites (47%) [149]. Factors such as high literacy levels, being 25 to 34 years old, engagement in sex work, and exposure to HIV intervention programs were associated with higher rates of HIV testing. Qualitative studies [147, 155] on HIV testing among MSM and TGW in two cities highlighted barriers such as HIV stigma and discrimination in healthcare settings and fears of adverse social consequences of testing HIV positive, and facilitators such as access to outreach programs operated by CBOs/NGOs, and accurate HIV risk perception.

Four studies (2 qualitative [144, 162] and 2 quantitative [148, 156]) conducted among MSM and TGW living with HIV reported that multilevel barriers prevented or significantly delayed access to free ART: the qualitative studies reported support from healthcare providers and peers as facilitators of ART adherence, while the quantitative studies [148, 156] indicated that 76% (n = 65/85) were on ART and 48% of these (n = 31/65) reported nonadherence [148]. Those who were younger and who had negative beliefs about ART were less likely to be adherent [148]. Low levels of knowledge, negative perceptions about ART, and ART nonadherence were significantly associated with lower levels of viral suppression [156].

In relation to access to gender-affirmative medical care, a qualitative study [154] reported a near-total absence of gender-affirmative hormone therapy and surgeries in public hospitals. Among three qualitative studies on challenges in accessing HIV testing and treatment services among MSM and TGW, two [157, 158] reported challenges faced by MSM and TGW in accessing HIV and gender transition-related services in the time of COVID-19.

### Interventions to improve health outcomes among LGBTQI+ populations

Eleven articles [165–175] focused on health-related interventions, especially in relation to HIV prevention, of which 10 were exclusively conducted with MSM. Six of the 12 studies were pilot studies, including four pilot RCTs [167, 171, 173, 175]. Two articles reported qualitative formative research studies to design counselling-based [166] and mobile phone-based interventions [170]. Studies of interventions to increase condom use or HIV testing utilized diverse modalities, such as face-to-face risk reduction counseling [167], provision of community-friendly services [168], virtual counseling [165], internet-based [175] and mobile phone-based messages [171], and motivational interviewing techniques [173, 174]. Other intervention studies used video-based technologies such as mobile game-based learning for peer education [172], and a video-based counseling session [165].

### New HIV prevention technologies and their acceptability

Overall, 18 studies [176–193] (11 quantitative, 7 qualitative) focused on new HIV prevention technologies, including oral pre-exposure prophylaxis (PrEP) [176, 177, 182, 184, 187–190, 192, 193], future HIV vaccines [178–181, 183] and rectal microbicides [185], as well as medical male circumcision [186], and oral HIV self-testing [191].

Of the ten articles on PrEP, eight examined acceptability or willingness to use PrEP among MSM and TGW; one explored the impact of prioritizing PrEP for MSM [184], and one compared the cost-effectiveness of offering PrEP to MSM with semiannual HIV testing as opposed to WHO-recommended 3-month testing [192]. Quantitative studies [176, 177, 188–190, 193] reported generally high willingness to use PrEP among MSM and TGW despite low levels of awareness. Qualitative studies [183, 188] reported factors associated with PrEP uptake, including perceived effectiveness in serodiscordant relationships, providing protection in cases of forced sex encounters, ability to use covertly, ability to have sex without condoms, and anxiety-less sex; barriers included PrEP stigma, fear of disclosure to one’s family or partners/spouse, and being labelled as HIV-positive or ‘promiscuous’ by peers. A mathematical modelling study [184] in Bangalore reported that PrEP could prevent a substantial proportion of infections among MSM (27% of infections over 10 years, with 60% coverage and 50% adherence).

Of the 5 studies on future HIV vaccine acceptability [178–181, 183], two [178, 180] assessed willingness to participate (WTP) in hypothetical HIV vaccine trials among MSM; one [179] explored mental models of candidate HIV vaccines and clinical trials; and two [181, 183] assessed frontline health service providers’ perspectives on HIV vaccine trials and their likelihood of recommending HIV vaccines to MSM populations.

### Underrepresented LGBTQI+ populations: Sexual minority women, transmasculine people and people with intersex variations

#### Sexual minority women

Seven studies (4%) focused on sexual minority women [194–200], while two additional studies [201, 202] included sexual minority women as part of a larger sample. Among the seven studies, most focused on romantic relationships, such as communication and prioritization in relationships [199], difficulties in maintaining relationships [196], understanding of intimacy [197, 198], and lack of legal recognition of same-gender romantic partnerships [198]. One study [197] used a collaborative ethnographic approach to capture the understanding of community and activism from the perspectives of “women loving women” which had indirect connections to mental health. Another study [200] documented resilience sources (for example, self-confidence, optimism) used by sexual minority women to cope with major stressors.

The sexual health of sexual minority women was explored in two studies [194, 198]. One used photo-elicitation interviews and a survey to explore health behaviors and concerns [194], reporting that a majority of sexual minority women were not accessing preventive healthcare services: 36% reported having been screened for breast cancer and 14% for cervical cancer, and only 20% had ever been tested for STIs. The other study [198] reported lack of knowledge regarding STIs and difficulty in identifying LGBTQ-friendly service providers as major barriers to accessing preventive services.

#### Transmasculine people

Two studies (1%) [203, 204] focused on transmasculine people’s health: one [203] documented challenges in negotiating gender identity in various spaces, such as family, educational settings, workplace and neighborhoods; and one [204] reported that a substantially higher proportion of transmasculine persons (36.3%) attempted suicide when compared with transfeminine persons (24.7%).

#### People with intersex variations

Among the two studies (1%) [205, 206] that focused on people with intersex variations, one [205] examined how healthcare professionals decide on gender assignment of intersex children, and the other study [206] documented the social stigma faced by people with intersex variations and their families. Findings from both of these studies highlighted that gender assignment decisions are influenced by sociocultural factors: parents of intersex children preferred a male gender assignment possibly because of the social advantages of growing up as a male in a patriarchal society.

## Discussion

This scoping review of a decade of peer-reviewed research on the health of LGBTQI+ people in India demonstrates a trend of increased publications addressing the health of sexual and gender minorities; however, it also identifies substantial gaps in the research—in terms of focal populations, geographical coverage, health conditions, and methods. Overall, this review demonstrates a predominant research focus on HIV and HIV-related risk behaviors among MSM and TGW populations; of these studies, a small subset were intervention studies aiming to improve the health of MSM and TGW. Notably, this review reveals the near complete omission of research on the health of sexual minority women—less than 4% of the studies identified. And amid the substantial focus on transgender women, largely in the context of HIV, scant research addressed the health of transmasculine people.

From a methodological perspective, among the quantitative studies that constituted the majority of the research, most were cross-sectional and descriptive in nature; few studies used longitudinal designs or mixed methods approaches, with very few intervention trials. The inclusion of a substantial proportion of qualitative and mixed methods studies, however, suggests a strength in the potential for characterizing the lived experiences of diverse LGBTQI+ people and experiences in the context of health disparities and challenges in healthcare access. Nevertheless, these too were dominated by a focus on MSM and TGW. A scoping review on LGBT inclusion in Thailand similarly reported substantial underrepresentation of lesbian and bisexual women, and transmasculine people, in the peer-reviewed literature [6].

The persistent and substantial gaps identified, even amid the overall increase in LGBTQI+ health research in India, have important implications for future research and research funding, health policies and programs, and healthcare services and practices for LGBTQI+ populations. There is a clear and compelling need to expand the evidence base on LGBTQI+ health in India to the many health and mental health conditions beyond HIV, and to the health challenges experienced across the diversity of LGBTQI+ people.

Specific population gaps identified in health research among LGBTQI+ people in India indicate the need for greater attention to lesbian and bisexual women, including potential health and mental health disparities compared to cisgender heterosexual women. Additional focus on lesbian and bisexual women’s experiences in access to and use of health services is sorely needed across an array of health conditions and healthcare settings, particularly given that studies reported their underutilization of routine preventive healthcare services. Reviews conducted on the health of sexual minority women in other countries arrived at similar conclusions [207, 208]. Further gaps emerged in the dearth of research with transmasculine people [209], and more broadly in research on access to medical and surgical gender-affirmative care needs for trans people. Greater attention to studies of healthcare providers and healthcare settings, and on healthcare provider training, that aim to improve access to gender-affirmative clinical services are needed [210]. Finally, there is a wholesale lack of health research among people with intersex variations. Future studies should focus on general health profiles, experiences in access to healthcare, and impact of non-essential or ‘corrective’ surgeries on health and mental health outcomes among people with intersex variations [211, 212].

Overall, the relatively small number of intervention studies were largely conducted with MSM in relation to HIV prevention. Nevertheless, while NACO supports several targeted interventions among MSM and TGW, with estimated programmatic coverage of nearly 88% to 95% of at-risk MSM and TGW [213], the lack of peer-reviewed publications on the effectiveness of such interventions limits their contribution to evidence-informed HIV prevention programs and policies in India. Although these interventions are primarily for programmatic purposes, the absence of published data represents a missed opportunity.

The stark lack of formal health outreach structures in India for lesbian and bisexual women, and for transmasculine people, makes it challenging to reach these populations through established organizational partners. Accordingly, greater involvement of a diversity of LGBTQI+ community-led groups in collaborative and participatory research studies is needed to expand opportunities to engage their inputs on research priorities, recruitment, and data collection methods, thereby also building their capacity in guiding and implementing research [214]. Such participatory mechanisms may be key to meaningful involvement of diverse and under-represented groups among LGBTQI+ communities and expanding relevant research evidence to advance their health. Strategic research funding mechanisms that target such underrepresented groups, as well as requiring community partnerships in certain health research streams, may be mechanisms to support such initiatives moving forward. For example, the U.S. NIH has established a sexual and gender minority research office, increased dedicated research funds, and released a five-year strategic plan to advance health research among sexual and gender minorities [215]. Similar steps need to be taken by the Indian Council of Medical Research, Department of Health Research. With just over one-fourth of the studies in this review funded fully or in part (in collaboration with NIH) by Indian government agencies, such as ICMR and NACO, there is a clear need to increase funding for LGBTQI+ health research by the Government of India.

This synthesis also highlights the connections between stigma, discrimination and violence, and the health issues faced by LGBTQI+ people. Several studies advance evidence on how discrimination and violence victimization contribute to psychosocial health problems and HIV risk among MSM and TGW [101, 216]. Stigma and violence elimination programs, and interventions in multiple sectors (for example, healthcare, education, employment) and social campaigns to promote understanding and acceptance of LGBTQI+ people are needed. The lack of access to gender-affirmative hormone therapy and surgeries for trans people highlights the need to improve access to such services, especially in the context of the Transgender Persons (Protection of Rights) Act, 2019, of India. This act clearly places the responsibility on the Indian central government and state governments to provide medical gender-affirmative health services and health insurance for trans people [22].

Other research areas that require increased exploration include the role of family and peer support in LGBTQI+ mental health, interventions to increase support from families and communities, and programs to eliminate discrimination and promote acceptance in healthcare, educational and workplace settings [25]. Given the deleterious impacts of stigma and discrimination on mental health and access to care, and the protective effects of social support and resilience resources, studies that integrate an understanding of social-structural contexts that affect mental health are key to effective approaches to advancing LGBTQI+ health [26]. Expanding the evidence base on LGBTQI+ health will require additional investments by national and state health research funders, including targeted funding for non-HIV-specific LGBTQI+ health research in the academic sector and in government-funded and government-run health programs on HIV (National AIDS Control Program of NACO), sexual and reproductive health and mental health (under National Health Mission), and non-communicable diseases (for example, National Program for Prevention and Control of Cancers, Diabetes and Cardiovascular Diseases and Stroke).

Finally, few studies made explicit reference to theoretical frameworks (for example, syndemic theory [216], minority stress theory [96], and structural violence [217]), that guided study design, analysis or interpretation. For one, such theories can advance research and understanding of the needs of understudied populations, such as sexual minority women, with studies also benefitting from community-based participatory methodologies and partnerships [198, 199]. The latter can advance application of theoretical frameworks that are sensitized to community-identified concerns, self-identifications, and priorities in Indian cultural contexts [199]. Several theoretical frameworks such as gender minority stress [218], gender affirmation [219] and intersectionality [220] that have been used productively in research among trans people in western countries, especially the U.S., appear not to be explicitly used in studies from India. Future research should include a focus on adapting existing frameworks to meaningfully address the Indian cultural context, as well as developing new indigenous frameworks for research with LGBTQI+ people in India. Future investigations should also ensure the inclusion of diverse subgroups of trans people—not solely gender binary, but also gender non-binary people—and portray local gender identity terms they use as well as indigenous constructions of gender identity, rather than defaulting to western terminologies, some of which do not translate well culturally or linguistically to the Indian LGBTQI+ experience [221].

### Strengths and limitations

This scoping review should be understood in the context of study limitations. First, we limited searches to English-language texts and those included in major academic databases; however, we are not aware of Indian native language-based academic journals, given that academics and researchers largely publish in English. Second, we did not conduct quality assessments of individual studies as this is outside the purview of a scoping review; we aimed to map the field of available research, and research gaps, rather than answer a specific research question [28]. Third, we limited our review to peer-reviewed articles, for which we identified a substantial number of sources. Future scoping or systematic reviews should include grey literature from across India to broaden understanding of the landscape of research and gaps in regard to LGBTQI+ health; this is particularly the case given the concentration of studies identified among a minority of Indian states, and conducted almost exclusively in urban areas. Further, we did not include asexual-identified people in this review; future reviews should include this subpopulation to understand their health needs and healthcare experiences [222].

## Conclusion

This scoping review identified key research gaps on LGBTQI+ health in India, with investigations largely limited to HIV-related issues, MSM and TGW populations, and urban study sites. This underscores the need for expanding health research in India to address the broad spectrum of LGBTQI+ people’s lives, specifically in moving beyond HIV-focused research to address mental health and non-communicable diseases as well. Future research should address the extensive gender gap in LGBTQI+ health research in India by focusing on health needs and healthcare experiences of lesbian and bisexual women. The broader spectrum of transgender and gender nonbinary people also merits increased focus, including studies on health needs and gaps with transmasculine people.

Finally, it is crucial to include sexual orientation and gender identity in national health surveys and to provide disaggregated data among LGBTQI+ subpopulations so that extant inequalities between heterosexual and cisgender people, and within LGBTQI+ people, can be documented [223]. Large-scale government-supported national health surveys among LGBTQI+ people provide a unique opportunity to document and explain health inequalities, and to identify potential solutions [224]. Strategies to enhance health research among LGBTQI+ people in India include developing a national LGBTQI+ health research agenda, providing dedicated LGBTQI+ health research funding from various government bodies, and investing in the training of researchers and new investigators to competently conduct LGBTQI+ health research. Additionally, investments in improving and sustaining the research and service provision capacities of community-based organizations are crucial as they already assume responsibility for serving a substantial number of LGBTQI+ people who are otherwise underserved by government-funded healthcare systems.

## Supporting information

S1 Checklist Preferred Reporting Items for Systematic reviews and Meta-Analyses extension for Scoping Reviews (PRISMA-ScR) Checklist. (DOCX)

S1 Appendix Sample search string for ProQuest database. (DOCX)
