# Correction: A scoping review of lesbian, gay, bisexual, transgender, queer, and intersex (LGBTQI+) people’s health in India

**DOI:** 10.1371/journal.pgph.0003644

**Published:** 2024-08-13

**Authors:** The PLOS GLOBAL PUBLIC HEALTH Staff

There are errors in the Competing Interest statement. The Competing Interest statement should read: The authors have read the journal’s policy and have the following competing interests: VC received partial salary support from the DBT/Wellcome Trust India Alliance Senior Fellowship at the time of this study. PAN serves on the Editorial Board for PLOS Global Public Health. This does not alter our adherence to PLOS Global Public Health policies on sharing data and materials.

The publisher apologizes for the error.
